# Red Light and Glucose Enhance Cytokinin-Mediated Bud Initial Formation in *Physcomitrium patens*

**DOI:** 10.3390/plants11050707

**Published:** 2022-03-07

**Authors:** Durga Prasad Biswal, Kishore Chandra Sekhar Panigrahi

**Affiliations:** 1 School of Biological Sciences, National Institute of Science Education and Research (NISER), Bhubaneswar 752050, Odisha, India; dpbiswal@niser.ac.in; 2 Homi Bhabha National Institute (HBNI), Training School Complex, Anushakti Nagar, Mumbai 400094, Maharashtra, India

**Keywords:** red light, blue light, glucose, bud initial, nutation, phytochrome, cryptochrome, cytokinin

## Abstract

Growth and development of *Physcomitrium patens* is endogenously regulated by phytohormones such as auxin and cytokinin. Auxin induces the transition of chloronema to caulonema. This transition is also regulated by additional factors such as quantity and quality of light, carbon supply, and other phytohormones such as strigolactones and precursors of gibberrelic acid. On the other hand, cytokinins induce the formation of bud initials following caulonema differentiation. However, the influence of external factors such as light or nutrient supply on cytokinin-mediated bud initial formation has not been demonstrated in *Physcomitrium patens*. This study deals with the effect of light quality and nutrient supply on cytokinin-mediated bud initial formation. Bud initial formation has been observed in wild type plants in different light conditions such as white, red, and blue light in response to exogenously supplied cytokinin as well as glucose. In addition, budding assay has been demonstrated in the *cry1a* mutant of *Physcomitrium* in different light conditions. The results indicate that carbon supply and red light enhance the cytokinin response, while blue light inhibits this process in *Physcomitrium*.

## 1. Introduction

In recent years the moss *Physcomitrium patens* (formerly *Physcomitrella patens*) has emerged as a major non-angiosperm plant model [1,2,3]. Mosses are considered to be among the earliest land plants. They predominantly exist as haploid gametophyte. The sporophyte is represented by a gametophyte-dependent spore-producing capsule for a shorter time. The gametophytic moss body has two developmental forms such as (i) protonema, a filamentous structure and (ii) gametophore, a miniature plant-like leafy structure. Moss spores on germination give rise to protonema. Protonema consists of chloronema: a chlorophyll-rich structure, with individual cells separated by a longitudinal cell wall; and caulonema: chlorophyll-deficient protonema cells with oblique cell wall. Chloronema are the first-formed cells upon spore germination. Caulonema arise from chloronema and they further give rise to bud initials or gametophore buds, which later produce the leafy gametophore [1]. In mosses these transitions are under the control of hormones. Classical plant hormones such as auxin, cytokinins (CKs), and abscisic acid (ABA) are present in mosses including *Physcomitrium* [4,5,6,7]. Presence of true gibberellic acid (GA) has not been reported in mosses; however, intermediates of the GA biosynthesis pathway have been identified in *Physcomitrium* [8,9]. In addition, the presence of strigolactones (SLs) and ethylene have also been reported [10,11]. Auxin enhances the transition of chloronema to caulonema [12,13,14,15,16]. This transition is partly influenced by SLs and GA-biosynthesis intermediates [8,17]. On the other hand, CKs induce branching of caulonema and formation of bud initials in different mosses [18,19,20,21,22,23,24]. ABA induces brood cell or brachycyte formation in mosses [25]. Ethylene regulates submergence- and osmotic stress-related responses in *Physcomitrium* [11,26]

Hormone signaling pathways are primarily regulated in response to environmental signals such as light. Light and hormone signals interact to regulate multiple developmental responses across the plant kingdom from algae to flowering plants [27,28]. Sunlight is a mixture of different wavelengths of light. However, plants primarily rely on specific wavelengths of light such as blue, red, and far-red (FR) to regulate the developmental responses. Plants perceive the quality, quantity, direction, and duration of light by different photoreceptors such as phytochromes (PHYs), cryptochromes (CRYs), phototropins (PHOTs), ZEITLUPE (ZTL), and UV resistance locus-8 (UVR8) proteins [29]. PHYs sense the ratio of red light (RL) and FR light (R:FR) in the environment and enable the plants to avoid shade or low light conditions [30,31]. On the other hand, CRYs, PHOTs, and ZTLs perceive blue light (BL) and UVR8 protein responds to ultra violet-B (UV-B) light [32,33,34,35]. Most of these photoreceptors (except PHOTs) upon activation by light, interact with transcription factors and modulate gene expression. These photoreceptors regulate multiple growth and developmental responses such as seed germination, seedling de-etiolation, shoot and root development, plastid development, chloroplast relocation, flowering, shade avoidance, phototropism, circadian rhythm, and photoperiodism [36,37,38]. The early land plant model *Physcomitrium* possesses all of these photoreceptors except ZTL [39,40,41,42,43]. Light regulates spore germination, protonema branching, phototropism, polarotropism, and chloroplast movement in *Physcomitrium* [44,45,46,47].

In higher plants light and hormone signals interact to regulate seed germination, hypocotyl elongation, flowering, fruit and root development, phototropic responses, and shade avoidance [28,48]. Light signals have also been shown to influence hormonal response in mosses. Higher amount of light enhances the chloronema–caulonema transition in *Physcomitrium*, which is an auxin-mediated process. This is primarily a photosynthetic effect, where the high energy condition enhances the caulonema differentiation [49]. Not only light quantity but light quality also influences this differentiation. Caulonema formation is enhanced under RL in *Physcomitrium*. On the contrary, BL inhibits this response through suppression of auxin-signaling components [40]. It appears that RL and BL regulate auxin response in opposing manners to balance the developmental transition of *Physcomitrium* in the natural environment [27].

While the chloronema–caulonema transition is a two-dimensional (2D) division, formation of bud initials, which are the precursors of leafy gametophores, marks the initiation of three-dimensional (3D) growth in mosses. This is an important event in the acquisition of land habitat and evolution of plant form. Bud initial formation, which is a CK-mediated process, has been reported to be influenced by light quality. In *Pohlia nutans* bud initial formation is induced under RL, but suppressed in BL [50]. The inhibitory effect of BL is partly suppressed when the protonema are cultured in kinetin-supplemented medium [51] or in a mixture of BL and RL [50]. In a study, protonema of *Funaria* produced lesser number of bud initials in response to exogenously supplied CK under RL and BL as compared to white light (WL). Interestingly, the protonema side branches showed curling under RL (nutations) [52], which may be the early signs of bud initial formation. Protonema, cultured in glucose- and CK-supplemented medium under dark, produced bud initials upon exposure to regular pulses of RL. This enhancing effect of RL was partly inhibited upon exposure of FR [52]. These facts indicate that RL enhances the bud initial formation, while BL and FR light inhibit the same in *Funaria*. In *Physcomitrium*, growth responses of protonema and gametophores are regulated by PHYs [44], however the role of light in regulating bud initial formation has not been demonstrated clearly. *Physcomitrium* has seven copies of PHYs such as PpPHY1-PpPHY4 and PpPHY5a-PpPHY5c [39] as well as two copies of CRYs such as PpCRY1a and PpCRY1b [40]. PHYs regulate cytoplasmic events such as phototropism, polarotropism, and chloroplast movement in protonema [40,46]. CRYs regulate protonema branching and phototropism in *Physcomitrium*. The *cry* mutants of *Physcomitrium* produce more gametophores under WL. This indicates that CRYs most likely inhibit bud initial formation [40]. However, it is not clear how CRYs play a role in CK signaling. These mutants have altered sensitivity to auxin signals [40], but it is not known whether the sensitivity or biosynthesis of CKs is altered in these mutants.

Apart from light quality, nutrient or carbon supply (energy status) also affect the growth and development of mosses. In *Funaria*, glucose has been shown to enhance CK-induced bud initial formation and this process in the dark was shown to be accelerated in presence of both sucrose and kinetin, but not in presence of sucrose alone [21]. This indicates that bud initial formation is not dependent on carbon supply, but it enhances the effect of kinetin which induces bud induction in the absence of light [53]. Later it was shown that glucose accelerated protonema growth and bud initial formation in *Funaria* in low irradiance light but had no significant effect in high irradiance light. In fact, more number of bud initials were formed under high irradiance light compared to low irradiance light, irrespective of the presence or absence of glucose [52]. *Ceratodon purpureus* showed earlier bud initial formation in medium supplemented with both sucrose and kinetin compared to medium with kinetin alone [22]. In *Physcomitrium turbinatum* bud initials have been shown to develop, even when the requisite light was provided in a discontinuous manner (divided into different combinations of light and dark periods). It has been hypothesized that light is responsible for synthesis of a morphogenic substance, which is required in optimum amount for bud induction. This substance accumulates over time in a cumulative manner under discontinuous light and induces bud formation upon reaching the optimum level [54]. Sugars are suitable and logical candidates for this purpose [7]. In fact, bud formation was delayed when the protonema of *Physcomitrium turbinatum* were cultured in medium without sugar [54]. Most of the earlier studies conducted to elucidate CK action in mosses have used carbon sources such as glucose or sucrose. It has been postulated that carbon sources may have an enhancing effect on CK-mediated bud formation in mosses [7]. However, carbon sources may also have a negative effect on the growth and development of mosses. For example, glucose has been reported to enhance protonema growth (primarily caulonema) in *Bryum billarderi*, but it inhibits the bud initial formation in presence of CKs [55]. Interestingly glucose enhances protonema development and shoot growth in *Bryum argentium*, but inhibits these processes in *Atrichum undulatum* [56].

Light quality and energy supply have been demonstrated to have a significant effect on auxin-mediated protonema differentiation in *Physcomitrium* [40,49], but the influence of these factors on CK-mediated bud initial formation has not been demonstrated. In this study we evaluated bud initial formation in wild type (WT) protonema with respect to glucose and light quality (WL, BL, and RL) using growth chambers equipped with light emitting diodes (LEDs) as light sources. In addition, we also evaluated the budding response in the *cry1a* mutant of *Physcomitrium* under RL, BL, and mixture of RL and BL (BR) to establish the role of BL in CK-mediated budding. Results suggest that while RL and carbon supply promote gametophore bud formation, BL inhibits this process in *Physcomitrium*.

## 2. Materials and Methods

### 2.1. Culture and Growth Conditions

WT strain of *Physcomitrium patens* (Hedw.) Mitt. and *cry1a* mutant [40] were used as plant material. Protonema cultures were maintained in modified Knop solid medium (250 mg KH_2_PO_4_, 250 mg KCl, 250 mg MgSO_4_·7H_2_O, 1000 mg Ca(NO_3_)_2_·4H_2_O, 12.5 mg FeSO_4_·7H_2_O, 12 g agar in 1000 mL, pH 4.5) [57,58] overlaid with cellophane disk (80 mm diameter, type 325 P, AA Packaging, Preston, UK) in 9 cm petri dishes by weekly sub-culturing. The culture medium was supplemented with Hogland’s A-Z trace element solution (1 mL/1000 mL Knop medium) (614 mg H_3_BO_3_, 389 mg MnCl_2_·4H_2_O, 110 mg Al_2_(SO_4_)_3_·K_2_SO_4_·24H_2_O, 55 mg CoCl_2_·6H_2_O, 55 mg CuSO_4_·5H_2_O, 55 mg ZnSO_4_·7H_2_O, 28 mg KBr, 28 mg KI, 28 mg LiCl, 28 mg SnCl_2_·2H_2_O, 25 mg Na_2_MoO_4_·2H_2_O, 59 mg NiCl_2_·6H_2_O in 1000 mL) [59]. One week old protonema tissues were ground with tissue homogenizer (Omni International, Kennesaw, GE, USA) and transferred to fresh Knop solid medium overlaid with a cellophane disk every week.

To observe the effect of light and glucose on bud initial formation, one week old protonema were ground and cultured in 100 mL liquid Knop medium (pH 4.5) in Erlenmeyer flasks capped with Silicosen^®^ silicone sponge plugs (Hirschmann Laborgeräte, Eberstadt, Germany). The ground protonema were grown under constant light (70 µmol·m^−2^·s^−1^) for four days (22 °C) in a photo-incubator shaker (Innova 44 Incubator Shaker, New Brunswick, Eppendorf, Hamburg, Germany) at 220 rpm. The four-day old protonema were used to observe the effect of light and glucose on CK-mediated bud initial formation.

Kinetin solution (Sigma-Aldrich, Catalag No. K0753) was prepared by dissolving it in 0.1 M NaOH. Four-day old WT protonema (grown in liquid medium under constant light) were cultured in liquid Knop medium (pH 5.8) supplemented with kinetin (1 µM) and without kinetin (control) in different light conditions such as WL, BL, and RL both in the presence of glucose (1%) (Sigma-Aldrich, Catalog No. G8270) or without glucose for two days in a long day (LD) condition (16 hr light/8 hr dark). Individual colonies were then observed and photographed in an Olympus IX73 or Zeiss Stereo Discovery.V20 microscope. Images were analyzed by Image J for counting the buds.

To study the role of BL in bud initial formation, WT and *cry1a* protonema from four-day old suspension culture were grown in liquid Knop medium (pH 5.8) with glucose (1%) in the presence or absence of kinetin (1 µM) for two days under WL, RL, BL, and a mixture of BL and RL (BR light). Individual colonies were then observed, photographed and analyzed as described earlier. The role of BL was further verified by culturing the WT protonema in BR light in the absence of glucose with or without kinetin. WT protonema were also cultured in FR light (in glucose containing medium with and without kinetin) to analyze its effect on bud initial formation.

The results were analyzed by Kruskal–Wallis ANOVA using GraphPad Prism (Version 9) or OriginPro 2019. Post hoc analysis was carried out using Bonferroni’s correction with a statistical significance of *p* < 0.05. The results were presented in median values using box plots.

### 2.2. Light Treatment

WL (50 µmol·m^−2^·s^−1^) was provided by plant growth chambers (Percival Scientific Inc. Perry, USA, Model No. CU36L6) equipped with fluorescent light, maintained at 22 °C. BL (30 µmol·m^−2^·s^−1^, 460 nm), RL (50 µmol·m^−2^·s^−1^, 670 nm), FR (50 µmol·m^−2^·s^−1^, 730 nm) and BR light were provided by LED chambers (Percival Scientific Inc. Model No. E-30LEDL3) maintained at 22 °C in a LD cycle. Light intensity and wavelength were measured by SpectraPen LM 510 (PSI Instruments, Drásov, Czech Republic).

### 2.3. Phytohormone Estimation

Seven-day-old protonema tissues cultured on cellophane disks were transferred to solid Knop medium (pH 5.8) supplemented with glucose and cultured for seven days in 16/8 hr light/dark cycle in WL. The tissue was harvested and snap-frozen in liquid nitrogen followed by grinding with liquid nitrogen. The ground plant material was then lyophilized (FreeZone 4.5, Labconco, Kansas City, MO, USA). *trans*-Zeatin (*t*Z) content was estimated according to Šimura et al. [60] (modified protocol). 25 mg of the lyophilized tissue was extracted in cold extraction buffer consisting of MeOH:H_2_O:HCOOH (15:4:0.1) with 25 mg *trans*-[^2^H_5_] Zeatin as internal standard. *t*Z was then purified and quantified by liquid chromatography coupled with triple-quadrupole-trap MS/MS (QTRAP 6500+ LC-MS/MS, SCIEX, Framingham, MA, USA). The experiment was repeated three times and data were presented as mean ± SEM. Phytohormone estimation was done in the metabolomics facility of the National Institute of Plant Genome Research (NIPGR), New Delhi, India.

## 3. Results

### 3.1. Gametophore Bud Formation Is Increased in Red Light and in Presence of Glucose

Kinetin-induced bud initial formation was observed in WT protonema of *Physcomitrium* under WL, BL, and RL in the presence and absence of glucose.

**Figure 1:**
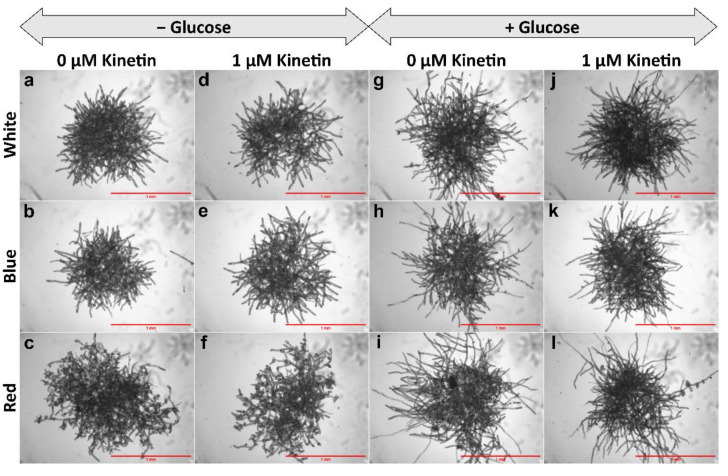
Morphology of protonema colonies under different light conditions. Protonema cultured in the presence of glucose show more growth (longer filaments) (**g**–**l**) compared to those in the absence of glucose (**a**–**f**). Protonema colonies cultured in red light in the absence of glucose do not show protruding filaments (**c**,**f**) as observed in other colonies, but show curling of filaments or nutations (peripheral regions). Scale Bar = 1 mm.

**Figure 2:**
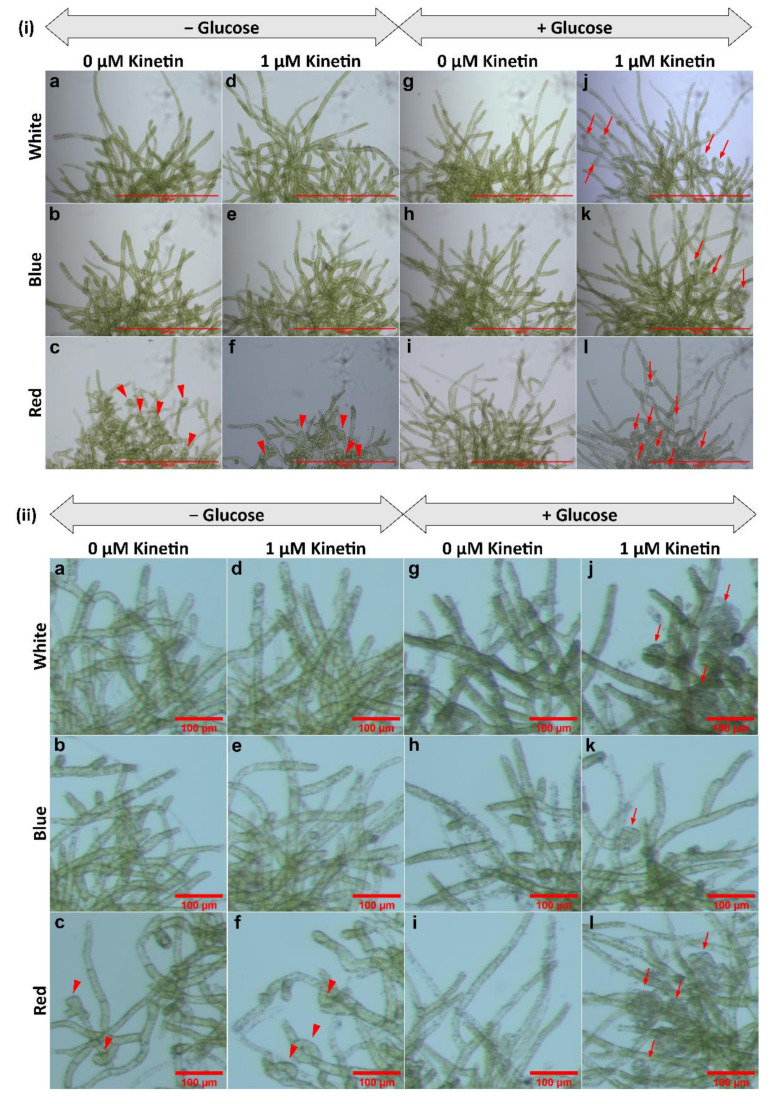
(**i**) Portions of protonema colonies showing the peripheral regions. Colonies under WL and BL show chloronema growth (green filaments). Colonies under RL show caulonema filaments (i and l) having less chlorophyll. Red triangles show the nutations and red arrows show the bud initials under RL. Scale Bar = 500 µm (0.5 mm). (**ii**) Portions of protonema colonies (magnified) showing presence or absence of nutations (red triangles) and bud initials (red arrows) under different light conditions. Nutations are present in RL in the absence of glucose, but bud initials are present in the presence of both glucose and kinetin. Scale Bar = 100 µm.

Bud initial formation was not observed in the controls (no kinetin), neither in the presence nor the absence of glucose (Figure 1a–c,g–i and Figure 2(i)a–c,g–i,(ii)a–c,g–i). No bud initials or only insignificant amounts of bud initials were developed in protonema cultured in the medium lacking glucose, but supplemented with kinetin in all light conditions (Figure 1d–f and Figure 2(i)d–f,(ii)d–f).

**Figure 3:**
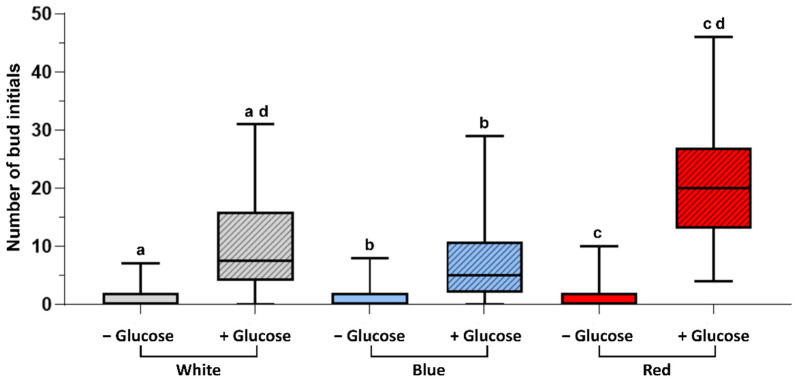
Box plots showing the comparison of bud initials formed in the presence and absence of glucose under different light conditions (in presence of 1 µM kinetin). In the absence of glucose very less number of bud initials or no bud initials are formed compared to their number in the presence of glucose. When glucose is present, maximum number of bud initials are formed in RL, which is significantly higher compared to the control (WL). Similar alphabetical letters indicate the significant difference between the bud initial numbers in the given condition (*p* < 0.05).

Gametophore buds were formed, when protonemata were cultured in the presence of both glucose and kinetin under all light conditions studied (Figure 2(i)j–l,(ii)j–l). Bud initial formation was observed to be much less in WL and BL. However, RL has a significant effect on bud initial formation and highest number of gametophores were formed under this light (Figure 3).

In all light conditions protonema growth was accelerated in the presence of glucose (Figure 1). However, under WL and BL, protonema showed predominant growth of chloronema irrespective of the presence or absence of glucose (Figure 2(i)a,b,d,e,g,h,j,k). On the other hand, protonema cultured under RL in the presence of glucose showed the emergence of caulonema filaments (Figure 2(i)i,l,(ii)i,l), but protonema cultured in the absence of glucose under RL did not show the emergence of caulonema filaments. Interestingly these protonemata (in the absence of glucose under RL) showed curling of newly-formed protonemal branches or nutations (Figure 2(i)c,f,(ii)c,f). The outline of these protonema colonies did not show protruding filaments due to curling of the protonemata (Figure 1c,f), which was observed in other conditions described earlier (Figure 1). Similar response has been described in *Funaria* under RL, but nutations were formed in presence of glucose, which is in contrast to *Physcomitrium*. These nutations later grew in a straight pattern in *Funaria* [52]. 

**Figure 4:**
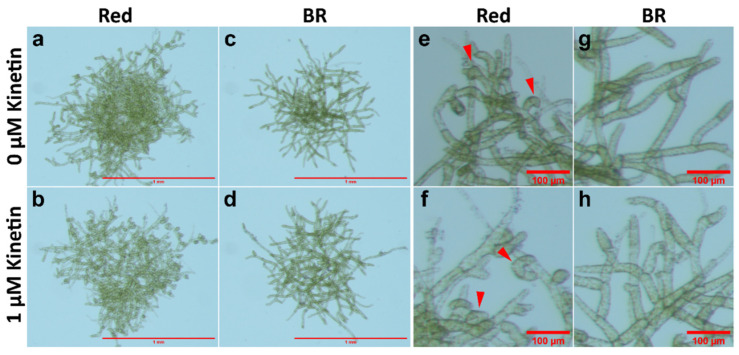
Comparison of WT protonema morphology under RL and BR light in the absence of glucose. **a**–**d** display complete protonema colony. **e**–**h** display magnified portions of protonema. Peripheral regions show nutations under RL (**a**,**b**,**e**,**f**). Nutations are not observed in BR light (**c**,**d**,**g**,**h**). Red triangles indicate the nutations (**e**,**f**). For **a**–**d**, scale bar = 1 mm. For **e**–**h**, scale bar = 100 µm.

The curling of protonema filaments was also tested in WT cultures under BR light in medium with and without kinetin (1 µM) but lacking glucose. The response was compared with cultures under RL. It was observed that in BR light, protonema did not show nutations (Figure 4).

### 3.2. cry1a Mutants Produce More Number of Gametophore Buds Than WT

**Figure 5:**
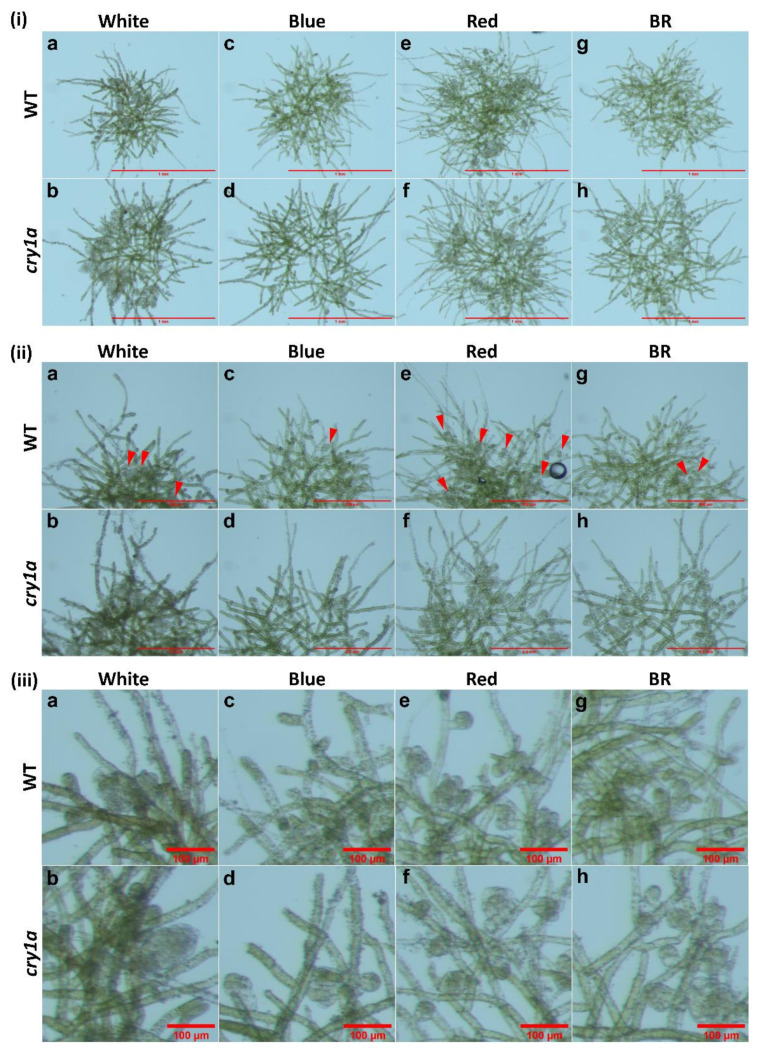
(**i**) WT and *cry1a* protonema colonies cultured in medium supplemented with glucose and kinetin (1µM) under different light conditions. In WT colonies bud initials are sparse (except RL) compared to *cry1a* plants, where these are visible (Scale bar = 1 mm). (**ii**) Peripheral regions of protonema colonies showing bud initials. Bud initials in WT plants are shown with red triangles. *cry1a* plants have more bud initials that WT plants. Scale bar = 500 µm (0.5 mm). (**iii**) Peripheral regions of protonema colonies (magnified) showing bud initials (Scale bar = 100 µm).

**Figure 6:**
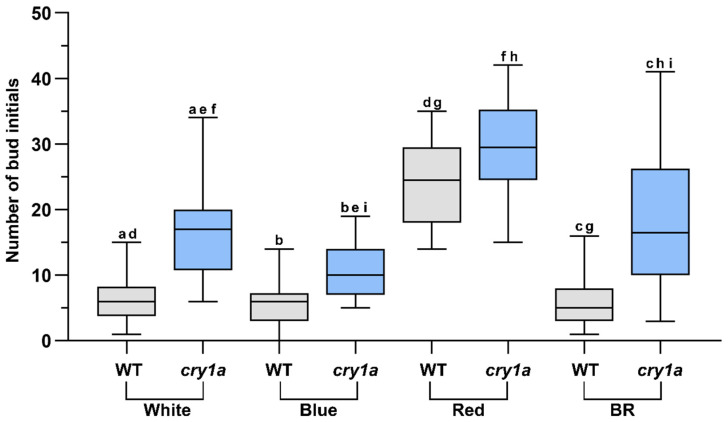
Comparison of number of bud initials in WT and *cry1a* protonema under different light conditions. Similar alphabetical letters indicate the significant difference between the bud initial numbers in the given condition (*p* < 0.05).

Bud initial formation was compared in WT and *cry1a* protonema in medium supplemented with glucose and kinetin under WL, BL, RL, and BR light (Figure 5). Under WL, BL, and BR *cry1a* protonema produced a significantly greater number of bud initials than WT. On the other hand, there was no significant difference in the number of buds produced between WT and *cry1a* mutants under RL (Figure 6).

WT plants showed no significant difference in the number of bud initials produced under WL and BR. A similar trend was observed in *cry1a* protonema, which also showed no significant difference in the bud initial numbers between WL and BR. On the other hand, while WT plants exhibit no significant difference in bud initial numbers under WL and BL, *cry1a* plants produced significantly greater number of bud initials under WL compared to BL. Both WT and *cry1a* protonema produced significantly greater number of bud initials under RL than BR light. When the bud initial numbers were compared between BL and BR, WT plants produced a comparable number of bud initials, but *cry1a* plants produced significantly higher number of bud initials under BR (Figure 6). Overall comparison indicates that *cry1a* plants produce more number of gametophore buds compared to WT plants.

### 3.3. Gametophore Buds Are Not Formed under FR Light

**Figure 7:**
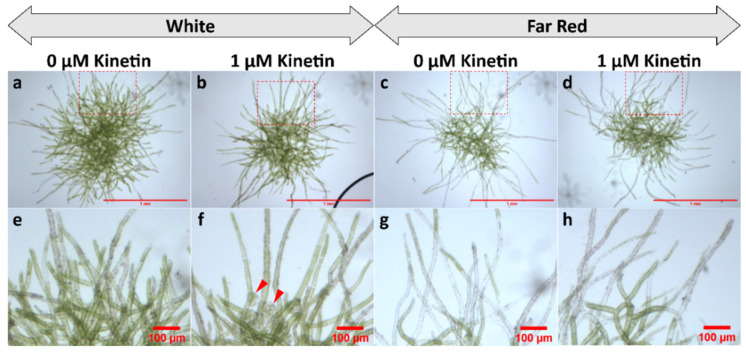
WT protonema colonies under WL and FR light in the presence of glucose. Upper panel shows the protonema colonies (**a**–**d**). Lower panel shows a portion of the peripheral region of the protonema colonies (magnified) (inset dashed red rectangle) displaying chloronema (**e**,**f**) or caulonema (**g**,**h**). Chloronemata are formed under WL (green filaments). Caulonemata are formed in FR light (less chlorophyll). Red triangles show the buds formed in WL in the presence of kinetin (**f**). For **a**–**d**, scale bar = 1 mm. For **e**–**h**, scale bar = 100 µm.

Protonema cultured under FR light (in glucose supplemented media) in presence or absence of kinetin did not develop bud initials. However, they showed predominant growth of caulonema filaments under FR irrespective of the presence of kinetin (Figure 7c,d,g,h).

### 3.4. Phytohoromone Estimation

**Figure 8:**
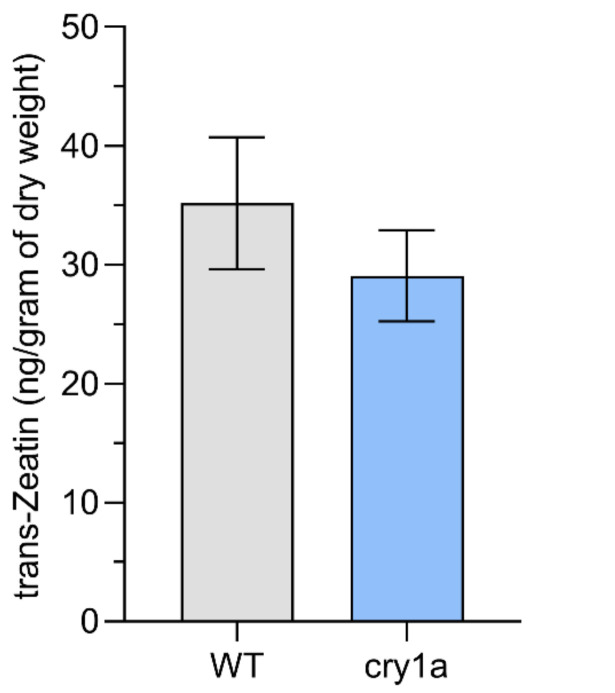
Comparison of *t*Z content in WT and *cry1a* plants. Comparison of tZ content in WT and cry1a plants. Comparison has been shown as mean ± SEM.

*t*Z content was estimated in WT and *cry1a* plants under WL. The difference between *t*Z content was not significant (Figure 8).

## 4. Discussion

### 4.1. Red Light and Glucose Enhance the Effect of Cytokinin

While light and carbon supply are known to influence auxin-mediated chloronema-caulonema transition in *Physcomitrium* [40,49], the influence of these factors on CK-mediated bud initial formation in *Physcomitrium* has not been clearly demonstrated.

In *Funaria* glucose induces CK-mediated gametophore bud initial formation [61]. In most of the previous studies involving CK response in mosses, carbon supply was provided either as glucose or sucrose. In the present study, bud initials were observed in WT protonema under WL, only when the culture medium was supplemented with both glucose and kinetin, but not in cultures lacking glucose and supplemented with kinetin alone. In the latter case either no bud initials were formed or rarely formed (Figure 2(ii)d–f). This indicates that optimum level of carbon source is required for CK response to initiate the bud initial formation.

Light quality also plays a role in the gametophore bud differentiation process. RL has been shown to have an enhancing effect on bud initial formation in different moss species. In this study, *Physcomitrium* also responded positively to RL as an inductive signal for bud initial formation, since the number of gametophore buds formed under RL was significantly higher compared to WL and BL (Figure 3 and Figure 6). 

**Figure 9:**
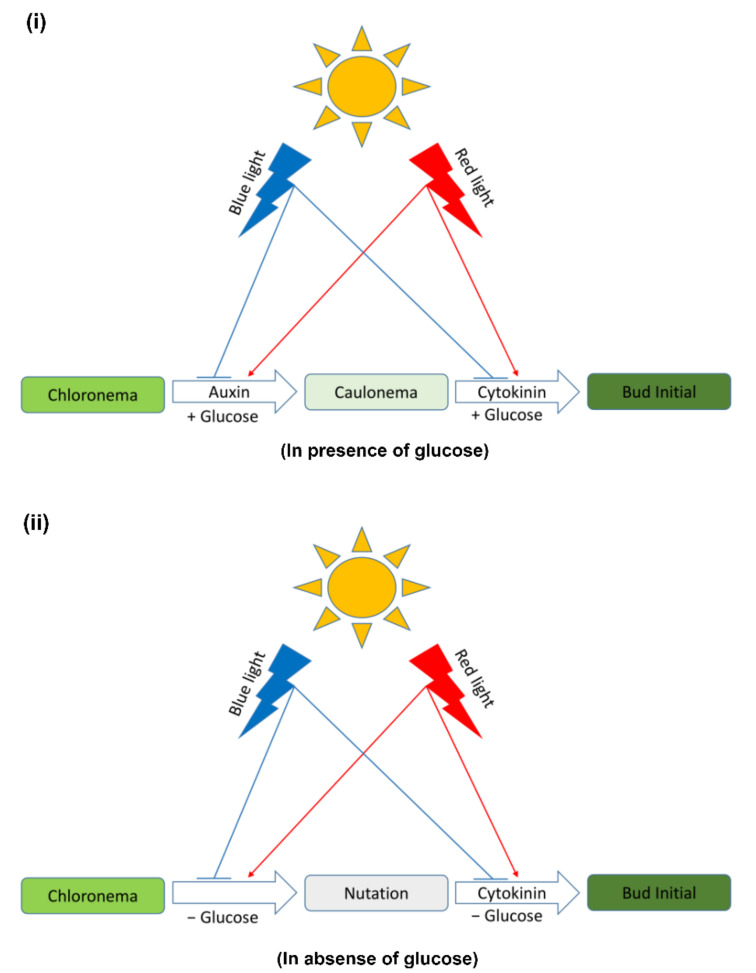
Graphical summary of the hypothesized mechanism of light and hormonal regulation of bud initial formation. (**i**) RL induces the formation of caulonema in presence of carbon sources such as glucose. Later bud initials are formed from caulonema branches. Glucose enhances the activity of auxin and CK. BL is inhibitory for the formation of both caulonema and bud initials (**ii**) RL induces formation of nutations in the absence of glucose. This process is inhibited by BL. Nutations later give rise to bud initials.

WT protonema predominantly produced chloronema filaments in WL and BL, and protonema growth was accelerated in presence of glucose. However, there was no sign of caulonema or bud initial formation in WL and BL in the presence or absence of glucose. On the other hand, protonema under RL showed differential response to the presence of glucose. It is known that caulonema formation is enhanced under RL in *Physcomitrium* [40]. In the present study, caulonema filaments were produced under RL in medium supplemented with glucose, but not in absence of glucose (Figure 1c,f,i,l and Figure 2(i)c,f,i,l,(ii)c,f,i,l). Protonema cultured in presence of both glucose and kinetin under RL produced bud initials. Nutations were observed under RL in medium lacking glucose. Even kinetin did not stimulate gametophore bud formation in this condition. This observation has two implications for the impact of RL on protonema differentiation. First, RL is the primary signal for bud initial formation. While gametophore buds are formed to a differential extent in WL, BL, and RL, nutations are observed only under RL. Nutations later develop into buds. Therefore, RL plays a prominent role in gametophore bud formation in *Physcomitrium*. Second, RL also requires an optimum amount of energy in the form of carbon supply to promote the activity of CKs. In presence of glucose, RL induces the formation of caulonema (instead nutation) followed by gametophore buds. The energy requirement may be an upstream step/pre-requirement for bud initial formation via the formation of caulonema filaments in the natural environment (Figure 9i). The absence of nutations in WL and BL indicates that BL may suppress the effect of RL to induce the formation of nutations in *Physcomitrium*. To test this assumption WT protonema were cultured in BR light in medium lacking glucose, but in presence and absence of kinetin. Interestingly nutations were not observed in BR light in absence of glucose and the protonemata were phenotypically similar to those under WL (Figure 1a,d and Figure 4). This indicates that BL inhibits the formation of nutations in natural environment under WL, when energy level is not optimum (Figure 9ii). Since nutations are formed in presence of kinetin, it appears that this process is independent of CK action. However, it is not clear that the formation of nutations is regulated by any other phytohormone such as auxin. Gametophore bud initials are formed later when the cells synthesize the optimum carbon sources required for CK activity (Figure 9ii).

### 4.2. *CRY1a* Suppresses Bud Initial Development

*Physcomitrium* possesses two copies of CRYs [40]. The double disruptant *cry1a cry1b* mutant of *Physcomitrium* has been shown to produce more number of gametophores than either *cry1a* or *cry1b* mutant and WT plants produce an even lower number of gametophores under WL [40]. This implicates their role in the inhibition of bud initial formation [40]. However, in the same study WT, *cry1a* and *cry1b* were shown to produce a comparable number of gametophores with the *cry1a cry1b* strain under BL [40]. Therefore, it is not clear whether the biosynthesis or sensitivity of CKs is inhibited. In the current study WT protonema developed a comparable number of bud initials under WL and BL in response to exogenously supplied kinetin. We therefore also compared the budding response of WT and *cry1a* plants to exogenously supplied kinetin under different light conditions. No significant difference was observed in bud initial numbers between WT and *cry1a* protonema under RL (Figure 6). Since RL-induced bud initiation is phytochrome-mediated, CRY1a may not have a major impact under monochromatic RL.

The difference in the formation of bud initials became apparent, when WT and *cry1a* strains were cultured under WL, BL, and BR. Under WL, which is a mixture of different light wavelengths, both PHYs and CRYs become active. The greater number of buds in response to exogenously supplied kinetin in the *cry1a* mutant indicates that CRY1a may play a role in sensing the CKs in vivo. The observations under WL were further established from the budding response under BR light, where a similar pattern was observed. Since in natural sunlight spectrum plants respond to BL and RL, BR light presents a condition similar to WL. Under BL *cry1a* plants develop more number of bud initials than WT plants. These facts indicate that CRY1a suppresses bud initial formation. *cry1* protonema also produced more number of bud initials under WL and BR light compared to BL, but these differences were insignificant in WT plants. This indicates that CRY1a endogenously inhibits the CK response by inhibiting the RL effect.

Different types of CKs such as *N* ^6^-(Δ^2^-isopentenyl)adenine (iP), *t*Z, *cis*-zeatin (*c*Z), dihydrozeatin (DHZ), and benzyl adenine (BA) are present in *Physcomitrium*. While cZ is the most abundant CK in *Physcomitrium*, iP, *t*Z, and BA display the most potent bud-inducing activity [6]. Since it is not known whether CRYs interfere with the sensitivity of the biosynthesis of CKs, *t*Z content was estimated in WT and *cry1a* plants cultured under WL. Both plants produce a comparable amount of *t*Z, but a differential amount of bud initials under WL. This indicates that CRYs may interfere with the CK-signaling. Biosynthesis and activity of other bioactive CKs might also be differentially regulated in *cry1a* plants of *Physcomitrium*. Since this study was conducted with a single CRY disruptant, there needs to be further investigation using double disruptant *cry1a cry1b* mutants to further establish the role of BL in CK-mediated bud formation.

### 4.3. FR Light Is Inhibitory for Bud Initial Development

No bud initials were developed in WT protonema in presence of kinetin. Protonema developed numerous caulonema under FR (Figure 7c,d,g,h), which may represent etiolated growth in protonema. It is known that caulonema formation is induced under RL [40], and it is required for bud initial formation. Caulonema formation also occurs under BL, but to a lesser extent compared to RL and it is almost absent under WL in standard culture conditions [40]. While overall growth is promoted under WL, BL, and RL, under FR fewer protonemata develop and more than 95% of the newly-formed protonemata are of caulonema type (unpublished data). This amount is much higher compared to RL as reported by Imaizumi et al. [40]. Calulonema formation has been shown to be induced under dark conditions and this shows red-FR reversibility where RL inhibits the dark-induced caulonema formation and FR light reverses this response [44]. While caulonema formation is followed by bud induction, bud initials were not developed in FR light even in the presence of glucose and kinetin (Figure 7). We observed that protonema cultured under FR light does not develop gametophores, but gametophores are produced (in lower numbers) when the protonema are pre-cultured in WL before their transfer to FR light (unpublished data). In WT plants of *Physcomitrium*, the number of gametophore bud initials decreases in a mixture of RL and FR light compared to RL, when the protonemata are cultured in kinetin-supplemented medium (unpublished data). These facts indicate that FR light is inhibitory for bud initial formation. Caulonema induction by RL and FR light perhaps represents two different developmental regulations mediated by auxin.

### 4.4. Light Quality and Carbon Supply May Influence the 2D–3D Transition in Physcomitrium

The evolution of 3D growth form is an important step in the colonization of land habit by plants. *Physcomitrium patens* is an excellent model to unravel the evolutionary innovations that facilitated the 2D–3D transition [62]. Since CKs induce the bud initial formation, the first 3D structures, they are important regulators of 2D–3D transition. The activity of CKs in plants are regulated by their synthesis, signaling, and degradation or inactivation [63]. ISOPENTENYLTRANSFERASEs (IPTs) are enzymes playing a major role in the rate limiting step of CK biosynthesis. *Physcomitrium* possesses seven copies of IPTs. *ipt1* knockout plants of *Physcomitrium* appear to have no defect in CK signaling, but show abundance of iP-type CK instead of *c*Z [64]. CK is perceived by CHASE domain-containing histidine kinases (CHKs). *Physcomitrium* encodes three CHKs namely PpCHK1, PpCHK2, and PpCHK3 [65]. *chk* mutants show a defect in gametophore development and differential budding in response to exogenous CK. Triple *chk* mutants show strong insensitivity to exogenous CK [65]. Cytokinin oxidases/dehydrogenases (CKXs) are enzymes catalyzing the degradation of CKs. In *Physcomitrium* the CKX gene family consists of six members [66]. Overexpression of *PpCKX1* results in delayed gametophore formation [66]. Since CK response is differentially regulated by light, the genes involved in CK biosynthesis, signaling, and degradation might also be differentially expressed under different light conditions.

In recent years numerous proteins have been identified which regulate the transition of 2D–3D growth downstream of CK signaling pathway in *Physcomitrium* [62]. These include CELLULOSE SYNTHASE 5 (CESA5), NO GAMETOPHORE 1 (PpNOG1), PpNOG2, DEFECTIVE KERNEL 1 (PpDEK1), AINTEGUMENTA, PLETHORA *and* BABY-BOOM (PpAPB), CLAVATA 3-like (CLE3) [62,67,68,69,70,71,72]. Some of these proteins are also regulated by auxin [62]. The mutants of these proteins show defect in gametophore bud formation.

We have shown that gametophore bud formation is regulated by light quality and energy supply. Therefore, it can be presumed that the components of CK response pathway as well as the proteins involved in 2D–3D transition might also be differentially regulated by these factors. Further studies are required to shed light on this aspect of CK response regulation.

## 5. Conclusions

*Physcomitrium* development has been shown to be regulated by the interaction of light and hormones. In addition, energy status also plays an important role in this differentiation process. BL and RL regulate the protonema differentiation process in an opposing manner, which is the growth and division in two dimensions. In this study we showed that light quality also influences the bud initial formation, which is the transition to 3D growth. Carbon supply appears to complement the effects of RL or PHYs. It is noteworthy that *Physcomitrium* possesses seven copies of PHYs. However, the role of individual PHYs in regulating the growth and development of *Physcomitrium* is not understood. We also showed that CRY1a may negatively regulate PHY action and thus CK response. Since *Physcomitrium* has another homolog of CRY1a, i.e., CRY1b, some of the responses which may be redundant, are difficult to interpret using the *cry1a* plants alone. Further studies involving higher order photoreceptor mutants are required to unravel the unknown aspects of light-regulation of the hormonal pathways in *Physcomitrium*.

## Data Availability

Not applicable.
